# Allergic asthma biomarkers using systems approaches

**DOI:** 10.3389/fgene.2013.00308

**Published:** 2014-01-08

**Authors:** Gaurab Sircar, Bodhisattwa Saha, Swati G. Bhattacharya, Sudipto Saha

**Affiliations:** ^1^Division of Plant Biology, Bose InstituteKolkata, India; ^2^Bioinformatics Center, Bose InstituteKolkata, India

**Keywords:** allergic asthma, biomarker, DAAB, TH-2 cytokines and ROS pathway

## Abstract

Asthma is characterized by lung inflammation caused by complex interaction between the immune system and environmental factors such as allergens and inorganic pollutants. Recent research in this field is focused on discovering new biomarkers associated with asthma pathogenesis. This review illustrates updated research associating biomarkers of allergic asthma and their potential use in systems biology of the disease. We focus on biomolecules with altered expression, which may serve as inflammatory, diagnostic and therapeutic biomarkers of asthma discovered in human or experimental asthma model using genomic, proteomic and epigenomic approaches for gene and protein expression profiling. These include high-throughput technologies such as state of the art microarray and proteomics Mass Spectrometry (MS) platforms. Emerging concepts of molecular interactions and pathways may provide new insights in searching potential clinical biomarkers. We summarized certain pathways with significant linkage to asthma pathophysiology by analyzing the compiled biomarkers. Systems approaches with this data can identify the regulating networks, which will eventually identify the key biomarkers to be used for diagnostics and drug discovery.

## Introduction

Asthma is a chronic immunological disorder of lung characterized by reversible airway obstruction, airway inflammation and increased airway hyperresponsiveness in response to provocative challenge. Physiological changes of the disease include the accumulation of inflammatory cells, especially the eosinophils, goblet cell metaplasia of lung epithelium with a mucus secreting phenotype (Laitinen et al., 1985). The worldwide incidence rate of asthma has been estimated to be 2.65 to 4/1000 per year and is more common among children with age less than 5 years where it ranges from 8.1 to 14/1000 per year (Gergen and Weiss, 1995). This case study also says that according to report presented by National Health and Nutrition Examination Survey (NHANES-2), this prevalence is higher in African-Americans (12.27) than in Caucasian (10.47) respectively. In Asia, adult asthma prevalence rate ranges from 3.6% in Hongkong, 2.4% in India, 0.1% in Singapore, 2.4% in Taiwan, and 2.91% in Thailand (Subbarao et al., 2009). Thus, asthma is arguably a major health problem worldwide deteriorating the quality of life of individuals affected and places a burden on their family and even the society. Indirect losses are due to disability, absenteeism and health care management. Focus of the present asthma biomarkers has been in the risk assessment before diagnosis, to determine the stage, grade of the disease during diagnosis and monitoring therapy or recurrent disease in the later stage of treatment. The biomolecules that undergo cellular, biochemical or molecular alterations in asthma patients vs. healthy subjects that are measurable in biological samples such as Broncho alveolar lavage Fluid (BALF), Nasal lavage fluid (NLF), blood or lung tissues may be considered as asthma biomarkers. These biomarkers are used for disease diagnosis and prognosis. A few native proteins that are targets for “hit” by a drug to achieve desirable therapeutic effects are another class of biomolecules which are known as “drug targets.” There has been a continuous quest for developing diagnostic biomarker to differentiate “allergic asthma” from other pulmonary inflammations and also to develop more biologic drugs by targeting biomolecules playing a key role in regulating asthma pathogenesis which may be more effective than traditional chemical drugs such as steroids (Murugan et al., 2009). Current research is focused on identifying key regulators and molecular pathways, which are associated in asthma pathogenesis. Systems approaches including genomics, proteomics, epigenomics and further integrating these attempts provide deeper understanding of the disease prognosis (Strimbu and Tavel, 2010). In this mini review, we gave a brief overview of different systems level approaches studied related to asthma biomarkers and we further focused on pathways, biological processes and molecular functions of these classes of biomarkers.

## Molecular biomarkers in allergic asthma

### Genomic approach

Genomic studies have reported large number of candidate biomarkers through both high and low throughput techniques. Experiments were done on human, mouse, monkey and rat model systems by comparing the expression of genes through challenging them with inhalant allergens and monitoring at different time intervals or by using resistant and susceptible strains of animals. Microarray based experiments reported hundreds of differentially expressed genes and hence plethora of information. Different samples like bronchial epithelial cells, eosinophils, CD4+ T-cells, mouse lung tissues have been employed in the experimental designs. The genes which showed significant differential expression were found to be linked with airway remodeling, production of mucus, macrophages and shifting the immune response toward Th2 phenotype thus enhancing asthma exacerbation (Laprise et al., 2004; Woodruff et al., 2007; Siddiqui et al., 2013). In most microarray experiments the differentially expressed genes were further validated either by RT-PCR or western blot. Genome Wide Association Study and Candidate gene approach have identified several regions on human chromosome which are linked to asthma phenotype. Nucleotide substitution in promoter region and ORF of IL4 receptor, IL13, HLA-II alleles, RANTES and CC-chemokine ligands were found to be strongly associated with asthma (Toda and Ono, 2002). We have compiled fifteen biomarkers from Database of Allergy and Asthma Biomarkers (DAAB)^1^ having more than two citations and listed in Table 1, out of which 11 were obtained from genomics.

**Table 1:** **List of asthma biomarkers cited in two or more times in Database of Allergy and Asthma Biomarkers (DAAB)**.

**Gene Symbol**	**Name of the genes/proteins**	**Sample**	**Organism**	**Approach**	**References**
ARG1	Arginase 1	BAL macrophages, BAL Fluid	Mouse, human	G^HL^, P^HL^	Siddiqui et al., 2013 [G^H^]
					Wu et al., 2005 [P^H^]
					Torrone et al., 2012 [P^H^
					Cloots et al., 2013 [G^L^]
					North et al., 2009 [P^L^]
BPIFA1	Palate lung nasal epithelial clone	BALFluid and nasal lavage fluid	Human	P^HL^	Wu et al., 2005 [P^H^]
					Ghafouri et al., 2006 [P^H^]
					Chu et al., 2007 [P^L^]
CPA3	Carboxypeptidase A3	Airway epithelial cells, bronchoscopy tissue sample	Human, mouse	G^HL^	Woodruff et al., 2007 [G^H^]
					Laprise et al., 2004 [G^H^]
					Balzar et al., 2011 [G^L^]
CCL8	Chemokine (C-C motif) ligand 8	Left lung tissue, BAL macrophages	Mouse	G^HL^	Park et al., 2008 [G^H^]
					Siddiqui et al., 2013 [G^H^]
					Fu et al., 2013 [G^L^]
Chi3l3	Chitinase 3-like3	BALFluid	Mouse	P^HL^	Greenlee et al., 2006 [P^H^]
					Zhao et al., 2005 [P^H^]
					Louten et al., 2012 [P^L^]
Chi3l4	Chitinase 3-like 4	BAL macrophages, BAL Fluid,	Human, mouse	G^HL^, P^HL^	Siddiqui et al., 2013 [G^H^]
					Webb et al., 2001 [G^L^]
					Greenlee et al., 2006 [P^H^]
					Zhao et al., 2005 [P^H^]
					Louten et al., 2012 [P^L^]
CLCA3	Calcium activated chloride channel -3	Airway epithelial cells, left lung tissue	Mouse	G^HL^	Woodruff et al., 2007 [G^H^]
					Park et al., 2008 [G^H^]
					Zhou et al., 2001 [G^L^]
Cxcl15	Chemokine (C-X-C motif) ligand 15	BAL Fluid	Mouse	P^H^	Greenlee et al., 2006 [P^H^]
					Zhao et al., 2005 [P^H^]
IL10	Interleukin 10	Lung tissue, CD4+T Cell	Mouse, human	rG^HL^	López et al., 2011 [G^H^]
					Hansel et al., 2008 [G^H^]
					Lyon et al., 2004 [G^L^]
IL13	Interleukin 13	CD4+T Cell,	Human	G^HL^, E^L^	Hansel et al., 2008 [G^H^]
					Durham et al., 2011 [G^H^]
					Kanoh et al., 2011 [G^L^]
MUC5AC	Mucin 5AC	Bronchoscopy tissue sample, Left lung tissue	Mouse	G^HL^	Laprise et al., 2004 [G^H^]
					Park et al., 2008 [G^H^]
					Ordonez et al., 2001 [G^L^]
NOS2A	Nitric oxide synthase	Bronchoscopy tissue sample	Mouse	G^HL^, E^L^	Laprise et al., 2004 [G^H^]
					Torrone et al., 2012 [E]
					Pascual et al., 2008 [G^L^]
Retnla	Resistin like alpha	Lung eosinophil, BAL macrophage	Mouse	G^HL^	Siddiqui et al., 2013 [G^H^]
					Tumes et al., 2009 [G^H^]
					Doherty et al., 2012 [G^L^]
SERPINB	Serpin peptidase inhibitor, clade B	Bronchoscopy tissue sample, airway epithelial cells	Human, mouse	G^HL^	Woodruff et al., 2007 [G^H^]
					Laprise et al., 2004 [G^H^]
					Karaaslan et al., 2012 [G^L^]
S100A9	Calcium binding protein A9	CD3+T cell	Human	P^HL^	Wu et al., 2005 [P^H^]
					Jeong et al., 2007 [P^H^]
					Lee et al., 2013 [P^L^]

G, Genomics; P, Proteomics; E, Epigenetics; BAL, Broncho alveolar lavage; H, High-throughput; L, Low-throughput.

In genomic studies of asthma several genes have been found to be significantly induced, of which some significant biomarkers are Chemokine ligands (CCL8, CCL5, CCL11, and CCL24), SERPINs (SERPINB2, SERPINB4, and SERPINA1) and CarboxypeptidaseA3. These three genes have not been studied earlier in detail however they have the potential of being used as asthma biomarkers. Chemokine ligands are potent attractants of Th2 lymphocytes at the site of lung inflammation in atopic asthma (Lukacs, 2001). SERPINS are members serine protease inhibitors family which inhibit neutrophil protease cathepsin G and mast cell chymase and protects the lower respiratory tract from damage caused by proteolytic enzymes. Thus, it can be used as potent diagnostic marker of asthma attack (Zou et al., 2002). Carboxypeptidase A3 is an asthma associated protease identified in lung epithelium and is a significant mast cell marker and was found to be upregulated in 42 non-smoking asthma patients (Woodruff et al., 2007). Retnla, also known as Fizz (found in inflammatory zone) protein is an inducible product of bronchial epithelial cell. This is considered as a marker of alternatively activated macrophages and highly polarized Th2 responses. In Retnla deficient mice the severity of atopic response is increased dramatically, whereas the IL13 response is suppressed by Retnla in airway hyper-responsiveness (Pesce et al., 2009). NOS2A is a gene that encodes inducible nitric oxide synthase, iNOS which produce nitric oxide (NO) from T lymphocytes in response to proinflammatory cytokines in an asthma model (Ricciardolo et al., 2004). This NO assists in the development of reactive nitrogen species such as peroxynitrites leading to cellular injury in the airways (Gabazza et al., 2003). NOS2A was found to be upregulated in bronchial biopsies in a microarray study (Laprise et al., 2004) and a (CCTTT)_n_ polymorphism in the promoter region was associated with asthma phenotype studied in White population (Pascual et al., 2008) and some SNP's were found on asthmatic children having Latino and Caucasian ancestry (Islam et al., 2010). These genes together with other mediators contribute to epithelial cell activation and dysfunction (Dougherty et al., 2010).

### Proteomic approach

Proteomic approaches are widely used to identify the expression level and modification of proteins to understand the pathophysiology of asthma. Proteomic signatures of lung parenchyma, BAL fluid, Immune cells (CD3+T cells or CD4+ T cells) from human or animal model have been used in different studies after experimental allergen challenge or after natural exposure to inhalant allergens. The advancement of proteomic techniques from earlier 2D gel based approach to recently more advanced LC-MS/MS based analysis resulted in precise identification of candidate proteins involved in asthma inflammation. In Table 1 we have listed six proteins identified in asthma proteomics studies, which can be analyzed in more detail to use them as clinical biomarkers. Similarly in asthma proteomics, a number of protein biomarkers have been identified, three of these potential biomarkers include AMcase (Chia, Chi3l3, Chi3l4, Chi3l1, and ChiT1), Calcium binding protein (S100A8 and S100A9), and Arginase (Arg1 and Arg2). These three proteins and their corresponding genes need further investigation at system level to reveal their use as potential diagnostic biomarkers.

AMcases are human chitinases induced via Th2 specific IL13 mediated pathway in aeroallergen challenged lung epithelium and macrophages as means of host defense. Th2 inflammation in asthma can be improved by targeted neutralization of these human chitinases (Zhu et al., 2004). A K(Lys)17R(Arg) polymorphism was identified in AMcase gene by genotyping study conducted on 322 pediatric asthma patients at University of Berlin and Freiburg (Bierbaum et al., 2005). Chi3l3 (Ym1) and Chi3l4 (Ym2) are other non-chitinolytic chitin binding proteins, have close linkage with asthma. Certain corticosteroids and leukotrienes receptor antagonist were shown to suppress the elevated pulmonary level of this protein (Zhu et al., 2004).

Calcium binding protein (S100A9/A8) form complex and inhibits macrophage activation and immunoglobulin synthesis by lymphocytes. Its homodimer also acts as a chemotactic agent for leukocytes and has pro-inflammatory activity on endothelial cell and inflammatory cells (Zhou et al., 2001). It is found in neutrophil cytoplasm and released upon cell activation (Cookson, 2002). This protein was found to be highly upregulated in endotoxin mediated response in non-smoking population challenged with endotoxin (Michel et al., 2013).

In asthmatic lung, Arginase expression is increased via Th2-induced, STAT6-dependent mechanism (Zimmermann et al., 2003). This affects arginine metabolism, and contribute to asthma pathogenesis through inhibition of NO generation and alterations of cell growth and collagen deposition (Shi et al., 2001). Association between four SNP's in this gene and atopic asthma were identified by genotyping 433 asthmatic case-parent triads in a public hospital of Mexican city (Huiling et al., 2006).

BPIFA1 (also known as SPLUNC), is highly expressed in the upper airways and nasopharyngeal regions and thought to be involved in inflammatory responses to irritants in the upper airways (Barnes et al., 2008). A Sialylated form of BPIFA1 was observed as post translational modification and was identified as being predominant in nasal lavage fluid (NLF) of allergy rhinitis patients (Ghafouri et al., 2006).

### Epigenomic approach

Epigenomics has emerged as a promising field, and have addressed the gaps in our current understanding of the interaction between nature and nurture in the development of asthma. Epigenetic modification can alter the DNA structure (by methylation, acetylation), the chromatin structure (by altering the Scaffolding protein) and by small non-coding RNAs. It was found that reduced Histone Deacetylase (HDAC) activity and increased Histone acetyl transferase (HAT) activity jointly promotes the expression of multiple inflammatory genes associated with asthma, however inhaled steroids reduce HAT activity to the normal level (Ito et al., 2002). External stimuli such as allergen exposure, cigarette smoke, traffic exhaust and folate rich diet cause methylation mediated silencing of genes like IFNγ, Fox-P3, IL2, iNOS and hypomethylation mediated activation of genes like IL6, IL4, IL8, and Acyl CoA thus increasing the Th2 phenotype assisting in the development of asthma (Durham et al., 2011). Usually IFN-γ and FOX-P3 undergo H4 acetylation and demethylation mediated activation to prevent post natal asthma and *in-utero* atopicity, respectively (Lovinsky-Desir and Miller, 2012). In the promoter region and other cis-acting element of two important Th2 cytokines like IL4 and IL13 demethylation causes recruitment of STAT6 and GATA3 thereby enhancing their expression (Miller and Ho, 2008). In addition to that small non-coding RNA plays a crucial role in fine epigenetic tuning of genes which are key factors in asthma pathophysiology (Durham et al., 2011). These include let-7, miR-9, miR-21, miR-125, miR-146a, miR-147, and miR-155. For example let-7 families of micro RNA and mi R-155 are found to inhibit expression of IL 13. This miRNA was found to block the IL13 R alpha 1 and ultimately lower the expression of STAT 6 thus controlling the Th2/Th1 balance in macrophages (Kumar et al., 2011 and Martinez-Nunez et al., 2011). An overexpression of miR21 and an underexpression of miR1 were demonstrated in IL-13 induced transgenic mice. This miR-21 was also found to control expression of IL12, a molecule responsible for Th2 mediated cellular response (Lu et al., 2009). A G/C polymorphism in miRNA146a gene locus resulted in a functional variant that in turn can significantly modulate expression of genes such as TNF-α, IL-6, Cox-2, iNOS, and RANTES that are closely linked with asthma pathophysiology (Jiménez-Morales et al., 2012). This polymorphism was found to have statistically significant association with a pediatric Mexican cohort.

#### Integrated approaches

**Figure 1:**
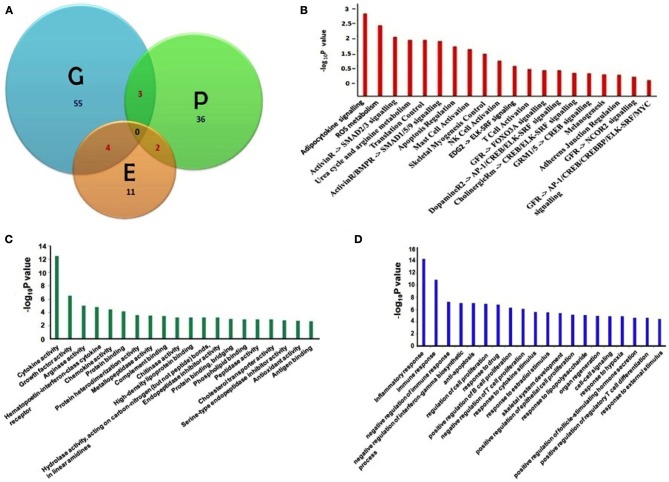
**(A)** Venn diagram showing asthma biomarkers identified in three different approaches of Genomics [G], Proteomics [P] and Epigenetic [E] studies with overlaps among the intersects. **(B)** Significant pathways; **(C)** enriched gene ontology molecular functions, and **(D)** biological function terms are listed which are linked with asthma biomarkers (Pathway and Gene Ontology analyses were done using Pathway studio 7.1^2^, Ariadane Genomics, Rockville, MD, USA). Pathways are significant where (−log_10_P) ≥ 1.3 (0.05% significance).

We have compiled the asthma biomarkers from different approaches including genomics, proteomics and epigenetics and have found little overlap amongst them as shown in Figure 1A. Detailed molecular information of all asthma related biomarkers are stored in DAAB. All the genes compiled from the high-throughput experiments have significant value (*p* = 0.05) of fold change, validated further by low-throughput techniques such as PCR, blotting and hold significantly close association with asthma pathophysiology.

Furthermore, we have listed fifteen genes in Table 1, which have been cited for two or more times in DAAB database. Asthma is dependent on many factors and thus it develops as a consequence of crosstalk among different pathways. Thus, we analyzed all the genes in our dataset compiled from several literatures in order to identify the pathways containing these biomarkers. Figure 1B shows cytokine pathways, ROS metabolism, NO metabolism and certain other metabolic pathways were significantly enriched (Detailed information of Figure 1B is shown in Table A1). In addition, Gene ontology of the biomarkers is shown in Figures 1C,D (Detailed information of GO terms are shown in Tables A2, A3). Cytokine activity, growth factor activity and Arginase activity were found to be significantly enriched in molecular function analysis. With respect to biological process inflammatory response, immune response and cell proliferation were found to be considerably predominating.

The most significant pathway triggering asthma has been the adipocyte signaling pathway. A few significant genes such as ACSL3, IL13, IL9, IL4, IL2, IL10, IFNA1, SOCS1, PON1, APOB, SOCS3, SCD, and NR1D1 were found to be the component of this pathway and associated with asthma pathogenesis (Tilg and Moschen, 2006; Diego et al., 2012). Adipokine or adipocytokine are cytokines secreted by the adipose tissues. These include Th2 cytokines and chemokines such as MCP1, RANTES, which are potent attractants of mast cells. There are also several clinical observations suggesting the role of obesity with asthma and one of the major conclusions so far has been the action of adipocytes derived cytokines which inhibit the activity of T-regs thus decreasing the tolerance (Theoharides et al., 2008). Cytokines such as TNFα, IL6 secreted by the adipocytes are important mediators of asthma. These molecules also affect vascular function by modulating nitric oxide and superoxide release. Some molecules such as leptin, adiponectin are the most abundantly expressed adipocytokines and are involved in classical cytokine pathway thus showing an asthmatic phenotype (Guzik et al., 2006).

Another significant pathway has been the ROS signaling pathway which is characterized by production of free radicals from molecular oxygen due to recruitment of activated inflammatory cells and associated with mitochondrial dysfunction that result in variety of physiological changes including increased airway reactivity, tissue injury and mucus production (Zuo and Clanton, 2005). Presently certain metabolites such as malondialdehyde, 8-isoprostane, exhaled NO, thiobarbituric acid are used as markers to measure the disease severity in sputum or exhaled air (Zuo et al., 2013). Several genes including MPO, PRDX6, SOD1, and CYBB as molecules involved in asthmatic responses and linked to ROS generation and hold the potential of using as biomarkers.

An additional significant pathway uncovered has been the Urea cycle and arginine metabolism. iNOS, ARG1, and ARG2 belong to this pathway and have also been found to be induced significantly in several genomic, proteomic, and epigenetic studies (North et al., 2009; Breton et al., 2011; Cloots et al., 2013). In asthmatic airway inducible NOS in inflammatory cells catalyses the production of NO from L-arginine, which results in the formation of reactive nitrogen species (RNS) that alters protein function by nitration of tyrosine residues thereby mediating inflammation and injury. In asthmatics upregulation of Arginase limits the availability of L-arg to iNOS thus generating peroxynitrite and concomitant nitration of proteins. It also enhances the level of L-ornithine which promotes airway remodeling by collagen deposition and excess cell proliferation (Ghosh and Erzurum, 2011).

## Conclusion

In the last few decades efforts to understand the pathophysiogy of allergic asthma has been intensified to a great extent because of increased mortality and morbidity. The aim of the present review is to focus on genes or their products which can be used as biomarker for allergic asthma. Occurrence of allergic asthma involves multiple genes, environmental factors and epigenetic mechanisms. Presently the potential difficulties to diagnose this disease are due to (i) remarkable overlap in symptoms of other pulmonary diseases, (ii) high interindividual and interpopulation variation at genetic level leads to changes in the uniformity of molecular marker, and (iii) absence of discriminative molecular markers, specific to atopic asthma, since most of the biomarkers currently used or in clinical trial are indicative of asthmatic inflammation irrespective of atopic background. Some of the common features of asthma exacerbation are eosinophilic inflammation, collagenitis, mucus deposition and extracellular matrix formation. However, these are common characteristics of other lung inflammations such as Chronic Obstructive Pulmonary Disease (COPD) or, non-allergic asthma. Therefore, the genes involved in these phenotypes may also be induced in all kinds of lung inflammations. To develop diagnostic markers exclusively for “allergic asthma” it is necessary to identify upstream components of the molecular pathways initiated immediately after allergen sensitization. Researchers can use these biomarkers for screening and risk assessment before the disease assumes severity by (i) identifying polymorphisms in wide population and (ii) correlating them with the alteration of signaling pathways that ultimately lead to allergic asthma. Since application of single biomarker approach to asthma research may not be realistic, newly identified biomarkers can be integrated in a multidimensional way to strengthen the treatment. Our mini review is focused on biomarker discovery by systemic approach using high-throughput “OMICS” platforms including genomics, proteomics and epigenetics and further some of them are well-studied in low-throughput experiments. Application of systems biology as a discipline provides a way to investigate the pathophysiology of asthma by giving a closer look to the system components, its dynamics and response to any kind of perturbation in the population level. Systemic approaches may emerge as a promising strategy to zoom into the global mechanism and identify features specific to asthma for developing better diagnostics and therapeutics.

### Conflict of interest statement

The authors declare that the research was conducted in the absence of any commercial or financial relationships that could be construed as a potential conflict of interest.

## Footnotes

^1^ http://bicresources.jcbose.ac.in/ssaha4/daab/.

^2^www.elsevier.com/online-tools/pathway-studio/biological-database

## Appendix

**Table A1:** **The list of pathways that play key role in asthma pathogenesis, as evident from the biomarkers identified by genomics, proteomics and epigenomics approaches**.

**Name of the pathway**	**Total entities**	**Expanded of entities**	**Overlap**	**Percent overlap**	**Overlapping entities**	***p*-value**	**(−log*P*)**
Adipocytokine signaling	52	780	13	1	ACSL3, IL13, IL9, IL4, IL2, IL10, IFNA1, SOCS1, PON1, APOB, SOCS3, SCD, NR1D1	0.001809	2.742605
ROS metabolism	43	74	3	4	PRDX6, SOD1, CYBB	0.004493	2.347436
ActivinR -> SMAD2/3 signaling	23	23	2	8	INHBA, INHA	0.010913	1.962064
Urea cycle and arginine metabolism	86	110	3	2	NOS2A, ARG1, ARG2	0.013536	1.868503
Translation control	86	984	13	1	CCL11, EGFR, IL13, IL9, IL4, IL2, IL10, CCL21, IFNA1, SOCS1, VEGFC, SOCS3, GNB2	0.013549	1.86809
ActivinR/BMPR -> SMAD1/5/9 signaling	27	27	2	7	INHBA, INHA	0.014876	1.827505
Apoptosis regulation	69	613	9	1	IL13, IL9, IL4, TGFB1, IL2, IL10, IFNA1, INHBA, INHA	0.022869	1.640747
Mast cell activation	64	529	8	1	PTGS2, IL13, IL9, IL4, IL2, IL10, IFNA1, ALOX15	0.027347	1.563092
Skeletal myogenesis control	70	569	8	1	EGFR, TGFB1, SOCS1, INHBA, CYBB, VEGFC, INHA, SOCS3	0.039923	1.39878
NK Cell Activation	59	523	7	1	IL13, IL9, IL4, IL2, IL10, IFNA1, TYROBP	0.067085	1.173375
EDG2 -> ELK-SRF signaling	33	78	2	2	EGFR, GNB2	0.102033	0.991259
T cell activation	81	1100	11	1	SGPP1, PTGS2, IL13, IL9, IL4, IL2, IL10, CTLA4, IFNA1, ALOX15, CNN1	0.129288	0.888442
GFR -> FOXO3A signaling	7	94	2	2	EGFR, VEGFC	0.138723	0.857852
DopamineR2 -> AP-1/CREB/ELK-SRF signaling	47	95	2	2	EGFR, GNB2	0.141106	0.850455
CholinergicRm -> CREB/ELK-SRF signaling	41	107	2	1	EGFR, GNB2	0.170351	0.768655
GRM1/5 -> CREB signaling	39	110	2	1	EGFR, GNB2	0.177823	0.750012
Melanogenesis	51	682	7	1	INMT, CCL11, EGFR, CCL21, PRDX6, VEGFC, GNB2	0.190612	0.71985
Adherens junction regulation	41	692	7	1	EGFR, TGFB1, INHBA, VEGFC, CDH11, INHA, DSP	0.20054	0.697799
GFR -> NCOR2 signaling	27	130	2	1	EGFR, VEGFC	0.228743	0.640652
GFR -> AP-1/CREB/CREBBP/ELK-SRF/MYC signaling	50	156	2	1	EGFR, VEGFC	0.296227	0.528375

*The data was generated using Pathway studio 7.1, Ariadane Genomics, Rockville, MD, USA. The column names are: Name of the pathway; Total entities; expanded entities; overlap; percent overlap; overlapping entities; p-value and −log_10_ p-value*.

**Table A2:** **The list of Gene Ontology Molecular Function (GOMF) terms that are significant in asthma pathogenesis, as evident from the biomarkers identified by genomics, proteomics and epigenomics approaches**.

**Name of the GOMF terms**	**Total entities**	**Expanded number of entities**	**Overlap**	**Percent overlap**	**Overlapping entities**	***p*-value**	**(−log*P*)**
Cytokine activity	217	217	13	5	Ccl8, Cxcl15, CCL11, IL13, IL9, IL4, IL2, IL10, CCL21, IFNA1, INHBA, INHA, SCGB3A1	3.30E–13	12.48204
Growth factor activity	198	198	8	4	IL9, IL4, TGFB1, IL2, INHBA, VEGFC, INHA, TFF2	3.10E–07	6.509308
Arginase activity	2	2	2	100	ARG1, ARG2	1.08E–05	4.967132
Hematopoietin-interferon-class (D200-domain) cytokine receptor binding	47	47	4	8	IL13, IL9, IL4, IFNA1	1.78E–05	4.750718
Chemokine activity	56	56	4	7	Ccl8, Cxcl15, CCL11, CCL21	3.57E–05	4.446842
Protein binding	7274	7274	41	0	Muc5ac, Serpinb3c, Apoa1, ACSL3, Igh-6, IGHG1, PTGS2, EGFR, ORM1, IL13, IL4, TGFB1, IL2, IL10, CTLA4, NOS2A, SERPINA1, SOCS1, SOD1, ARG2, APOB, CYBB, TIMP4, FCGR2B, POSTN, FOXP3, CDH11, INHA, S100A9, SOCS3, TYROBP, VIM, TCF21, FBN1, C4BPA, AATF, SCNN1G, HSPA1B, ITIH1, LCN1, GNB2	7.37E–05	4.132694
Protein heterodimerization activity	268	268	6	2	EGFR, TGFB1, INHBA, APOB, CYBB, INHA	0.000265	3.576101
Metallopeptidase activity	178	178	5	2	CPA4, MMP12, ADAM33, ACE, ADAM8	0.000314	3.502791
Complement binding	9	9	2	22	CFB, C4BPA	0.000383	3.417369
Chitinase activity	11	11	2	18	Chi3l3, CHIA	0.000582	3.235176
High-density lipoprotein binding	11	11	2	18	Apoa1, PON1	0.000582	3.235176
Hydrolase activity, acting on carbon-nitrogen (but not peptide) bonds, in linear amidines	11	11	2	18	ARG1,ARG2	0.000582	3.235176
Endopeptidase inhibitor activity	118	118	4	3	SERPINA1, SERPINB2, CSTA, ITIH1	0.000639	3.194323
Protein binding, bridging	60	60	3	5	DSP, CSTA, COL11A1	0.001039	2.983192
Peptidase activity	633	633	8	1	Slpi, CPA4, MMP12, SERPINA1, ADAM33, ACE, CFB, ADAM8	0.001171	2.93128
Phospholipid binding	64	64	3	4	Apoa1, PON1, APOB	0.001254	2.901838
Cholesterol transporter activity	16	16	2	12	Apoa1, APOB	0.001256	2.901021
Serine-type endopeptidase inhibitor activity	150	150	4	2	Serpinb3c, SERPINA1, SERPINB2, ITIH1	0.001558	2.807388
Antioxidant activity	20	20	2	10	PRDX6, SOD1	0.001972	2.705175
Antigen binding	79	79	3	3	Igh-2, Igh-6, IGHG1	0.002296	2.63899

The data was generated using Pathway studio 7.1, Ariadane Genomics, Rockville, MD, USA. The column names are: Name of the GOMF terms; Total entities; expanded entities; overlap; percent overlap; overlapping entities; p-value and −log_10_ p-value.

**Table A3:** **The list of Gene Ontology Biological Process (GOBP) terms that are significant in asthma pathogenesis, as evident from the biomarkers identified by genomics, proteomics and epigenomics approaches**.

**Name of GOBP terms**	**Total entities**	**Expanded number of entities**	**Overlap**	**Percent overlap**	**Overlapping entities**	***p*-value**	**(−log*P*)**
Inflammatory response	293	15	5	5	Ccl8, Cxcl15, CCL11, PTGS2, ORM1, IL13, IL9, IL4, TGFB1, IL10, NOS2A, CCL21, CYBB, ALOX15, S100A9	4.54E–15	14.34325
Immune response	604	16	2	2	LILRA6, Ccl8, Cxcl15, IGHG1, CCL11, IL13, IL9, IL4, IL2, IL10, CTLA4, CCL21, FCGR2B, CFB, C4BPA, CHIA	1.24E–11	10.90786
Negative regulation of immune response	14	4	28	28	TGFB1, CTLA4, FCGR2B, FOXP3	5.90E–08	7.229314
Negative regulation of interferon-gamma biosynthetic process	4	3	75	75	INHBA, FOXP3, INHA	8.79E–08	7.055796
Anti-apoptosis	198	8	4	4	IL2, IL10, SOD1, ALOX15, SOCS3, SERPINB2, AATF, HSPA1B	9.69E–08	7.013485
Regulation of cell proliferation	135	7	5	5	PTGS2, EGFR, TGFB1, NOS2A, ADAM33, INHA, SCGB3A1	1.18E–07	6.929752
Response to drug	295	9	3	3	Apoa1, PTGS2, MMP12, TGFB1, SOCS1, SOD1, CYBB, TIMP4, SOCS3	1.62E–07	6.791126
Positive regulation of B cell proliferation	23	4	17	17	Igh-6, IL13, IL4, IL2	5.12E–07	6.290996
Negative regulation of T cell proliferation	26	4	15	15	TGFB1, IL10, CTLA4, FOXP3	8.58E–07	6.066354
Response to cytokine stimulus	77	5	6	6	PTGS2, SERPINA1, SOCS1, TIMP4, SOCS3	2.73E–06	5.563052
Response to estradiol stimulus	79	5	6	6	PTGS2, TGFB1, ERPINA1, SOCS1, SOCS3	3.11E–06	5.50781
Skeletal system development	147	6	4	4	TGFB1, INHBA, POSTN, CDH11, INHA, FBN1	3.98E–06	5.400395
Positive regulation of epithelial cell proliferation	44	4	9	9	EGFR, MMP12, TGFB1, VEGFC	7.50E–06	5.125108
Response to lipopolysaccharide	99	5	5	5	PTGS2, SERPINA1, SOCS1, TIMP4, SOCS3	9.43E–06	5.025503
Organ regeneration	49	4	8	8	Apoa1, TGFB1, SOCS1, SOCS3	1.16E–05	4.936449
Cell–cell signaling	275	7	2	2	IL13, IL2, IL10, CCL21, INHBA, INHA, S100A9	1.35E–05	4.869351
Response to hypoxia	184	6	3	3	TGFB1, NOS2A, SERPINA1, ACE, SOCS3, SCNN1G	1.44E–05	4.842389
Positive regulation of folliclE-stimulating hormone secretion	3	2	66	66	INHBA, INHA	2.38E–05	4.62283
Positive regulation of regulatory T cell differentiation	3	2	66	66	IL2, FOXP3	2.38E–05	4.62283
Response to external stimulus	23	3	13	13	INHBA, PON1, INHA	3.75E–05	4.426548

The data was generated using Pathway studio 7.1, Ariadane Genomics, Rockville, MD, USA. The column names are: Name of the GOBP terms; Total entities; expanded entities; overlap; percent overlap; overlapping entities; p-value and −log_10_ p-value.
